# Genome analysis of new *Blattabacterium* spp., obligatory endosymbionts of *Periplaneta fuliginosa* and *P*. *japonica*

**DOI:** 10.1371/journal.pone.0200512

**Published:** 2018-07-10

**Authors:** Cláudia S. L. Vicente, Shakhinur Islam Mondal, Arzuba Akter, Sota Ozawa, Tasei Kikuchi, Koichi Hasegawa

**Affiliations:** 1 NemaLab/ICAAM—Instituto de Ciências Agrárias e Ambientais Mediterrânicas, Departamento de Biologia, Universidade de Évora, Núcleo da Mitra, Évora, Portugal; 2 Department of Environmental Biology, College of Bioscience & Biotechnology, Chubu University,Matsumoto, Kasugai, Aichi, Japan; 3 Division of Parasitology, Faculty of Medicine, University of Miyazaki, Miyazaki, Japan; 4 Genetic Engineering and Biotechnology Department, Shahjalal University of Science and Technology, Kumargaon, Sylhet, Bangladesh; 5 Biochemistry and Molecular Biology Department, Shahjalal University of Science and Technology, Kumargaon, Sylhet, Bangladesh; University of Minnesota, UNITED STATES

## Abstract

The successful adaptation of cockroaches is, in part, dependent of the activity of their obligatory endosymbionts, *Blattabacterium* spp., which are involved in uric acid degradation, nitrogen assimilation and nutrient provisioning. Their strategic localization, within bacteriocytes in the proximities of uric acid storage cells (urocytes), highlights their importance in the recycling of nitrogen from urea and ammonia, end-products not secreted by their host insects. In this study, we present the complete genome sequence of two new *Blattabacterium* spp. from *Periplaneta fuliginosa* (BPfu) and *P*. *japonica* (BPja), and detailed comparison with other *Blattabacterium* strains from different cockroach species. The genomes of BPfu and BPja show a high degree of stability as showed with for other *Blattabacterium* representatives, only presenting a 19-kb fragment inversion between BPja and BPfu. In fact, the phylogenomics showed BPja as an ancestor species of BPfu, BPLAN (*P*. *americana*) and BBor (*Blatta orientalis*), in congruence with their host cockroach phylogeny. Their functional profile is similar and closest to the omnivorous strain BBge (*Blattella germanica*). Interesting, BPja possesses the complete set of enzymes involved sulfate assimilatory pathway only found in BBge and BMda (*Mastotermes darwiniensis*). The newly sequenced genomes of BPja and BPfu emphasise the remarkable stability of *Blattabacterium* genomes supported by their long-term coevolution and obligatory lifestyle in their host insect.

## Introduction

Considered “living-fossils”, cockroaches (order Blattodea) are one of the most primitive insect group, dating back as far as the Late Carboniferous (320 Mya) [1]. They can inhabit a wide range of ecosystems, from tropical forests to deserts, coping with extremes and food scarcity by using varied combinations of movement, habitat choice, physiological mechanisms and lifecycle strategies [2]. Cockroaches are mostly considered omnivores or generalists, and are able to select their diet according to their nutrient demands at every stage in their lifecycle [2]. Although set with sophisticated physiological mechanisms to resist and prevail under stressful conditions (more detail in 1), much of these insect’s success is correlated with the close relationships established with their microbiome.

The microbiome of insects comprehends in a consortium of microbial communities colonizing the external and internal body structures, and which are involved in a wide range of important lifestyle functions such as colonization and resistance to parasites and/or pathogens, diet breakdown, nutrient recycling and production of pheromones and/or kairomones [3]. The diversity of these communities is host-depended and is mainly determined by its habitat, diet, developmental stage and phylogeny [4]. Apart from these microbial inhabitants, primary endosymbionts are particularly interesting since they live solely within specialized host cells, undergo vertical transmission to offspring and contribute extensively for host health and development [5]. These endosymbionts include the most highly constrained, stable and specialized symbioses known in the animal world [5]. Cockroaches harbour the endosymbiont *Blattabacterium* (phylum Bacteriodetes, class Flavobacteria) in their abdominal fat body, inside specialized cells (mycetocytes or bacteriocytes), which are adjacent to urocytes (cells that store uric acid) [6]. Early studies showed that aposymbiotic cockroaches (cockroaches reared with antibiotic-containing food) were less fitness that normal cockroaches, showing a smaller size, light color, reduced fecundity and increased development time [7]. These observations emphasised the importance of the endosymbionts to their roach host. *Blattabacterium* spp. are involved in nitrogen recycling from ammonia and urea, and subsequently the provision of essential amino acids and vitamins [8]. In fact, the location of the endosymbionts suggests dynamic metabolic interaction between the bacteriocytes and the urocytes [9].

The host-dependent lifestyle of *Blattabacterium* has effect on their genome composition, size and structure. Till date, several genome sequences of *Blattabacterium* have been annotated from different cockroaches and their phylogenetic closest termites: *Blatella germanica*, BBge [10]; *Periplaneta americana*, BPLAN [8]; *Cryptocercus punctulatus*, BCpu [11]; *Blaberus giganteus*, BGIGA [12]; *Mastotermes darwiniensis*, BMda [13]; *Nauphoeta cinerea*, BNCIN [14]; *Blatta orientalis*, BBor [15]; and *Panesthia angustipennis*, BPAA [16]. Here, we present the complete genomes of two *Blattabacterium* spp. isolated from the cosmopolitan *P*. *fuliginosa* and the endemic *P*. *japonica*, both cockroaches considered serious pests in Japan with contrasting environmental adaptations, and provide insights into the evolutionary stability of the endosymbionts genomic and metabolic architecture.

## Materials and methods

### Cockroach strains and rearing

*Periplaneta fuliginosa* EE and *P*. *japonica* Miyoshi strains are established in the Hasegawa Laboratory (Chubu University, Japan) since 2013, under the conditions described by Vicente et al. [17].

### Total DNA extraction from fat body tissue and quantification of *Blattabacterium*

One adult male of each cockroach strain was surface-cleaned with 70% EtOH and dissected using sterile forceps and tweezers. The fat body tissue was carefully removed and weighed (*P*. *fuliginosa* EE fat body weighing = 181.9mg; *P*. *japonica* Miyoshi fat body weighing = 118.8mg). Total DNA extraction of fat body tissue was performed using DNeasy Blood & Tissue kit (Qiagen, USA, California) with some modifications to the original manufacturer’s instructions. After weighting, the fat body was transferred into ATL buffer, homogenized with a sterile hand-held homogenizer, and incubated for 1h at 55ºC with 20µL of Proteinase K solution (540 units/mL, Wako). The homogenate was filtered through a 0.22µM diameter filter (Millipore) and the filtrate used for DNA extraction (next steps in the kit protocol). The quality and quantity of the DNA samples was measured using NanoVue plus spectrophotometer (GE Healthcare Life Science, USA). DNA samples were stored at -20ºC.

Host DNA was extracted from the muscle tissue of each cockroach strain using DNeasy Blood & Tissue kit (Qiagen, USA, California) following the manufacturer’s instructions. The quality and quantity was assessed as above mentioned. The quantification of *Blattabacterium sp*. in the fat body of each cockroach strain (BPful, *Blattabacterium sp*. from *P*. *fuliginosa*; and BPjap, *Blattabacterium sp*. from *P*. *japonica*) was inferred by the ratio between the DNA host sample and the respective DNA endosymbiont sample. For both, a standard curve (log DNA concentration plotted against Cq-values) was determined by quantitative real-time PCR (qPCR) using single copy genes, urease *ureA* (for *Blattabacterium sp*.) and wingless *wg* (for *Periplaneta sp*.) [18]. The DNA concentrations tested ranged between 0.63-10ng/µL for the endosymbiont, and 2-50ng /µL for the host. *ureA* primers were designed based on the multiple sequence alignment (MSA) of *ureA* gene of all *Blattabacterium spp*. with known genomes (Table 1): universal *ureA*_for (5’-ATG CAY TTA AMT TYT TAT GAA-3’) and universal *ureA*_rev (5’-TCA TAT WKY YRT ATT YTC TYT TWC C-3’). MSA was conducted with default parameters in BioEdit version 7.2.5 using ClustalW. *wg* primers designed for *Periplaneta genus* were: *wg*_for (5’-TGG TCT ACT TGG AGC CTT CC-3’) and *wg*_rev (5’-ATC CAC GCC TAT CGA CGT AT-3’). qPCR was performed using CFX96TM Real-Time (Bio-Rad), and SYBR Premix Ex TaqTM II (Tli RNAse H Plus) kit (Takara Bio Inc., Japan) following the manufacturer’s indications. The thermal cycling conditions were: initial denaturation at 95ºC for 30 secs; 39 cycles of denaturation at 95ºC for sec, annealing and extension at 60ºC for 30 secs; followed by the melting curve. For accuracy, the standard curve was three times repeated and the efficiency calculated using the equation (1) PCR efficiency = (10^(-1/S)^-1) × 100, where S is the slope of the standard curve.

**Table 1:** Characterization of BPfu and BPja genomes in comparison with other published *Blattabacterium* strains.

Strain	BPfu	BPju	BNCIN	BGIGA	BBge	BPLAN	BBor	BPAA	BCpu	Bmda
**Genome size (bp)**	645 082	636 644	626 627	632 588	640 335	640 442	638 184	632 490	609 561	590 554
**Plasmids**	1	1	1	1	1	1	1	*	1	1
**Plasmid size (bp)**	4127	3781	3675	3423	3485	3448	3735	*	3816	3306
**Chromosome size (bp)**	640 955	632 863	622 952	629 165	636 850	636 994	634 449	*	605 745	587 248
**G + C content (%)**	28.1/30.8	27.6/29.7	26.2/20.6	25.7/30.9	27.1/29.8	28.2/28.5	28.2/30.6	26.4/-*	23.8/30.5	27.5/31.9
**Total number of genes**	640	623	627	616	631	634	627	624	589	597
**Total CDS**	590 + 6	572 + 6	581 + 5	573 + 4	586 + 4	587 + 4	572 + 7	575*	545 + 3	544 + 4
**rRNAs**	3	3	3	3	3	3	3	3	3	3
**tRNAs**	34	34	32	34	34	33	33	34	32	34
**Other ncRNAs**	1	0	1	1	3	1	3	3	3	3
**Pseudogenes**	6	8	5	1	1	6	9	9	3	9

Host species abbreviations and accession numbers: BPfu, *Periplaneta fuliginosa*; BPja, *Periplaneta japonica*; BNCIN, *Nauphoeta cinereal* (NC_022550.1); BGIGA, *Blaberus giganteus* (NC_017924.1); BBge, *Blattella germanica* (NC_013454.1); BPLAN, *Periplaneta americana* (NC_013418.2); BBor, *Blatta orientalis* (NC_020195.1); BPAA, *Panesthia angustipennis* (NC_020510.1); BCpu, *Cryptocercus punctulatus* (NC_016621.1); BMda, *Mastotermes darwiniensis* (NC_016146.1). The following features result from the analysis of both bacterial chromosome and plasmids (if present): genome size, number of base pairs, total number of genes, G+C content and total CDS. The asterisk (*) indicates no information given by the authors of the original study [16].

### Sequencing, assembly and annotation

Paired-end sequencing libraries were prepared from 100 ng of endosymbiont enriched DNA using the Nextera DNA Library Prep kit (Illumina) according to the manufacturer’s instructions. Libraries were sequenced on the Illumina MiSeq sequencer with the v3 kit (301 cycles x 2). The raw sequence data were analysed using the RTA 1.12.4.2 analysis pipeline and were used for genome assembly after removal of adapters, low quality, and duplicate reads to produce a total of 2.5 and 4.2 Gb sequence data for *Blattabacterium* from *P*. *fuliginosa*; and *P*. *japonica*, respectively. Illumina sequence reads were assembled using the Platanus assembler version 1.2.4 [19] with options (assembly; -k 91, scaffolding and gapclosing; default parameters). The Platanus assemblies were further improved using MITObim and manually curated using gap5 [20]. The assembled genomes were annotated using the Prokaryotic Genome Annotation System (PROKKA) [21] and the Rapid Annotations using Subsystems Technology (RAST) server [22]. The annotations were, then, manually curated using *in silico* Molecular Cloning software (*In-silico* biology, https://www.insilicobiology.jp). Raw sequences, genome assembly and gene annotation were deposited in DDBJ (DNA Data Bank of Japan) under BioProject PRJDB6862 (*Blattabacterium* sp. *P*. *japonica*, SAMD00114544 and DRX119986; and *Blattabacterium* sp. *P*. *fuliginosa*, SAMD00114545 and DRX119985).

### Functional categorization and pathway reconstruction

The protein-coding genes in the genome were subjected to COG analysis using WebMGA [23] and IMG [24]. The COG profile was displayed using the ‘Heatmapper’ web tool [25]. KEGG website, BlastKOALA and KEGG Mapper, was used for metabolic pathway reconstruction [26]. Re-examination and verification of the pathways were performed according to the pathway descriptions in the EcoCyc [27] and MetaCyc [28] databases.

## Results and discussion

To ensure a complete genome coverage of both BPful and BPjap, an estimation of ratio host: endosymbiont DNA from the total fat body DNA was determined using independent standard curves for host (*wg* gene) and endosymbiont (*ureA* gene) (S1 Fig). The ratio of *P*. *japonica*: BPjap was 1:3 and *P*. *fuliginosa*: BPful was 1:1.8. These ratios indicated that both endosymbionts genomes were 3x BPjap and 1.18x BPful represented in total host DNA.

### Genome characteristics

The genomes of primary endosymbionts reveal a high degree of stability as a result of convergent patterns of evolution such as genome reduction and AT-richness [5]. The genomes of the newly sequenced *Blattabacterium* genomes from *P*. *fuliginosa* (BPfu) and *P*. *japonica* (BPja) also show the same trend in comparison with other *Blattabacterium* (Table 1). The BPfu genome is 645,082 bp in length and is composed of a 640,955 bp chromosome and a 4,127 bp plasmid, with a GC content of 28.1% and 30.8%, respectively. The BPfu chromosome encodes 634 protein-coding genes, including 6 pseudogenes, 3 rRNA genes, 1 ncRNA gene, and 34 tRNA genes assigning all 20 standard amino acids (S2 Table). The pseudogenes were *metG* (methionine-tRNA ligase), *lolD* (outer membrane-specific lipoprotein transporter subunit), *mvaK* (mevalonate kinase), *ygaD* (ABC transporter ATP-binding protein) and two genes coding for hypothetical proteins, and are involved in protein synthesis, lipoprotein translocation, isoprenoid/sterol synthesis and multidrug export respectively. The BPja genome is 636,644 bp in length consisting of a 632,863 bp chromosome and a 3,781 bp plasmid. The GC content of chromosome and plasmid is 27.6% and 29.7%, respectively. The BPja chromosome encodes 618 protein-coding genes, distributed as follows: 8 pseudogenes, 3 rRNA genes, and 34 tRNA genes assigning all 20 standard amino acids (S3 Table). The BPja pseudogenes were *gltX* (glutamyl-tRNA synthetase), *atpG* (ATP synthase, gamma subunit), *lolD*, *dnaN* (DNA polymerase III), *resP* (resolvase) and three genes coding for hypothetical proteins, which are related, respectively, with protein synthesis, ATP synthesis, lipoprotein translocation, DNA synthesis and protein degradation.

Both BPfu and BPja plasmids, named pBPFU1 and pBPJA1, respectively, encode 6 protein-coding genes: *nrdB* (ribonucleotide-diphosphate reductase subunit beta), *dut* (deoxyuridine triphosphatase), and four genes coding for hypothetical proteins, which belong to metabolic pathways for DNA replication and nucleotide biosynthesis. The same genes were also identified in BNCIN [14]. In general, the number of genes in the *Blattabacterium* plasmids varied between 3 to 7 and also share a high identity between *Blattabacterium* from different host species [14].

### Phylogenomics and gene synteny

**Fig 1:**
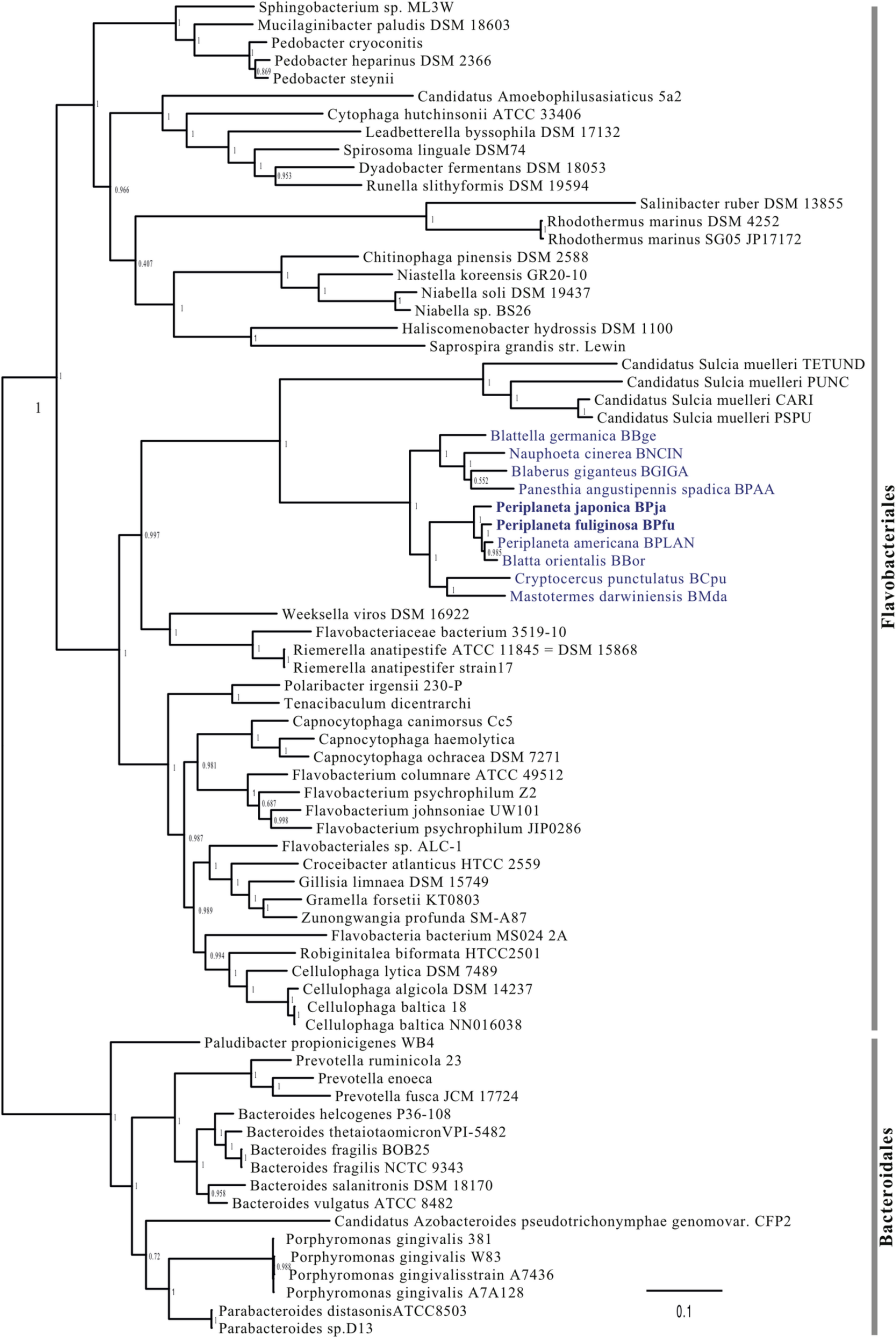
Phylogenomics analysis of 75 Bacteroidetes including *Blattabacterium* strains.

The phylogenetic analysis between BPfu and BPja and other endosymbiont representatives of phylum Bacteroidetes is presented in Fig 1. Within Flavobacteriales order, all *Blattabacterium* strains are grouped in the same clade and organized in different three sub-clades. One sub-clade groups BBge and BNCIN, BGIGA and BPAA. Another sub-clade is composed by the *Blattabacterium* strains from wood-feeding cockroach *C*. *punctulatus* (BCpu) and primitive termite *M*. *darwiniensis* (BMda). The third and monophyletic sub-clade groups BPja, BPfu, BPLAN and BBor, all *Blattabacterium* strains from omnivorous and worldwide pest cockroaches. The phylogenies of endosymbionts and their hosts are generally congruent evidenced by their long-term evolution [10, 29–30]. In this study, the topology of this third sub-clade shows BPja as an ancestor species of BPfu, BPLAN and BBor, which was also seen in the topology of their host cockroach [31].

**Fig 2:**
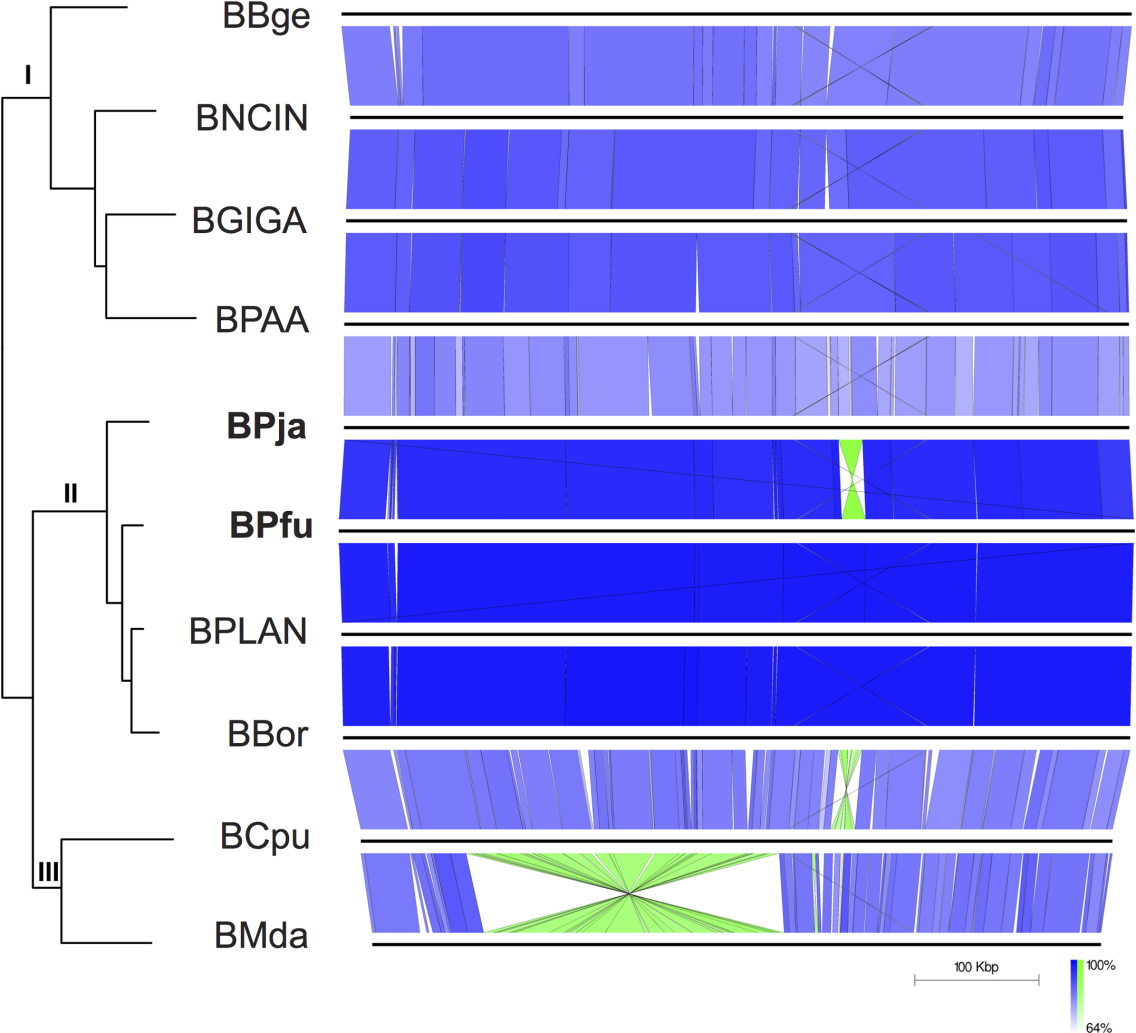
Gene order comparison between all *Blattabacterium* strains. Lines between genomes connect orthologous genes in blue if genes are in the same orientation, or in green if they are inverted. The first gene in all strains is *yidC* (membrane protein insertase C).

The intragenomic changes are quite rare in most primary endosymbionts [5, 8, 15]. The synteny between all sequenced *Blattabacterium* genomes (Table 1) is presented in Fig 2. The newly sequenced BPja and BPfu genomes reinforce the remarkable stability, in terms of genome structure and content, of the other *Blattabacterium* genomes. Only the *Blattabacterium* BCpu and BMda show more divergence compare to other sequenced *Blattabacterium* genomes [11, 13, 15]. Within the sub-clade II, one inversion occurred, a 19-kb fragment between BPja and BPfu (Fig 2). In clade III, another inversion occurred between BBor and BCpu (20-kb fragment), already denoted by other authors [11, 13, 15] (Fig 2). Other recombination events, such as the presence of large and repetitive intergenic regions observed in the obligate endosymbiont *Portiera* from whitefly host (*Trialeurodes vaporarium*) [32], were absented suggesting the general model for genome evolution of gene inactivation and subsequent loss [33–34].

### Functional profile and metabolic pathways

**Fig 3:**
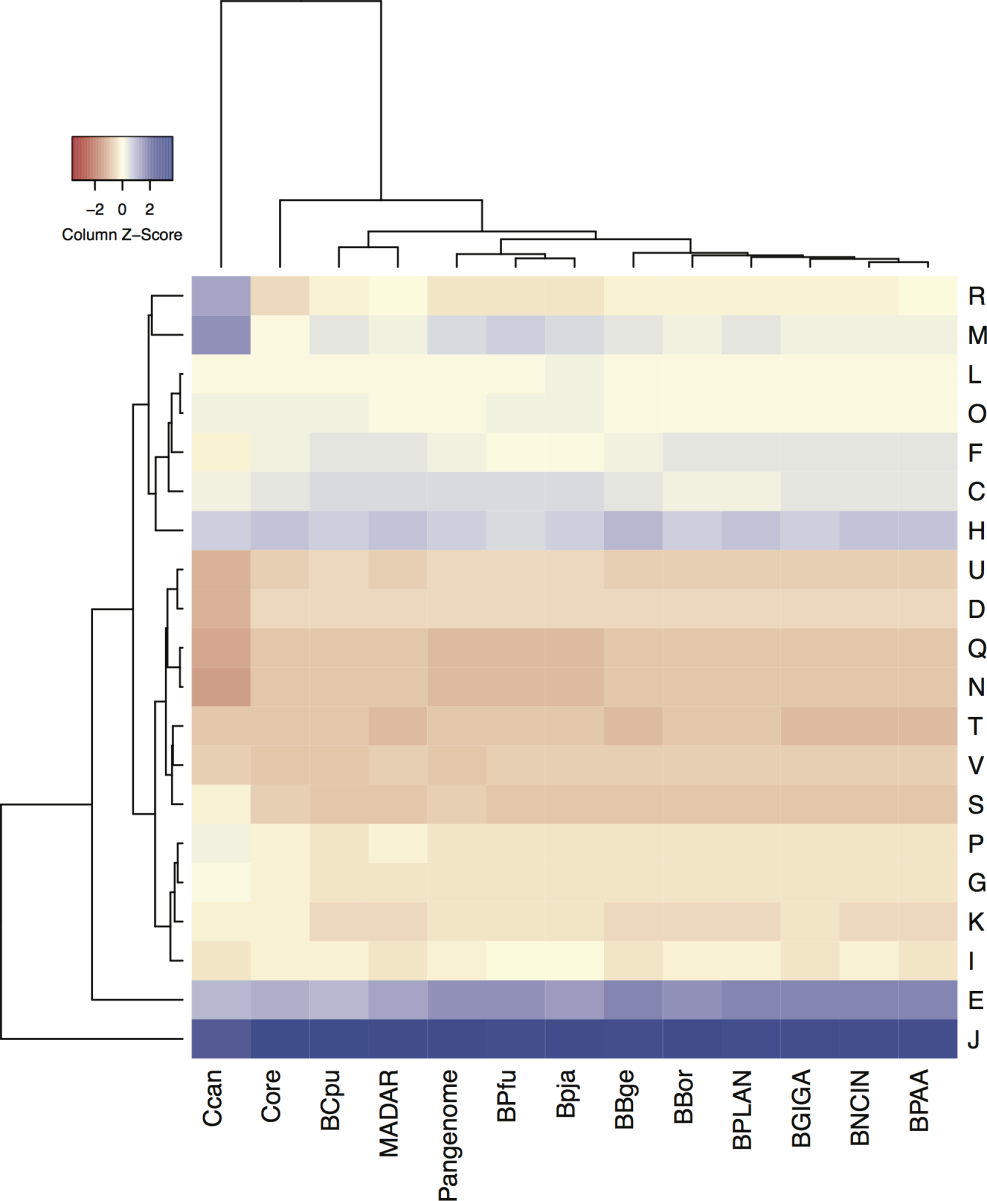
COG frequency heat map of different *Blattabacterium* strains with their pan- and core-genome, and the free-living *Capnocytophaga canimorsu*. By alphabetic order: C, energy production and conversion; D, cell cycle control; E, amino acid transport and metabolism; F, nucleotide transport and metabolism; G, carbohydrate transport and metabolism; H, coenzyme transport and metabolism; I, lipid transport and metabolism; J, translation; K, transcription; L, replication, recombination, and repair; M, cell/wall membrane biogenesis; N, cell motility; O, post-translational modification, protein turnover, chaperones; P, inorganic ion transport and metabolism; Q, secondary metabolites biosynthesis, transport, and catabolism; R, general function prediction only; S, function unknown; T, signal transduction mechanism; U, intracellular trafficking and secretion; and V, defense mechanism.

Selective pressure across insect hosts and historical contingencies are responsible by specific changes in gene content and pathways among closest endosymbiont strains [35]. The functional profile of BPja and BPful and the other *Blattabacterium* strains, categorized in COG, are presented in Fig 3 and S4 Table. Notwithstanding, the clustering diagram with the *Capnocytophaga canimorsu* as outgroup, shows the functional profile of BPfu and BPja are closer to the pan-genome and the omnivorous strains, BBge, whereas the strains from the two wood-feeding species, BCpu and BMda, cluster with the core genome. Several expected features arose from this analysis, such as the predominance of genes in COG category J (translation) within the core and it is the most represented functional category in the endosymbiont genomes. This was expected as most of the conserved universal COGs are contained within this category [36]. The second most represented functional category, especially in the omnivorous species, contains genes involved in amino acid transport and metabolism (E) and shows the main gap between the wood-feeding species and the others, because it harbours most of the gene losses described in these two strains [11, 13]. The exception is in other wood feeding species BPAA which is close to BNCIN and BGIGA as described by Tokuda et al. [16].

**Fig 4:**
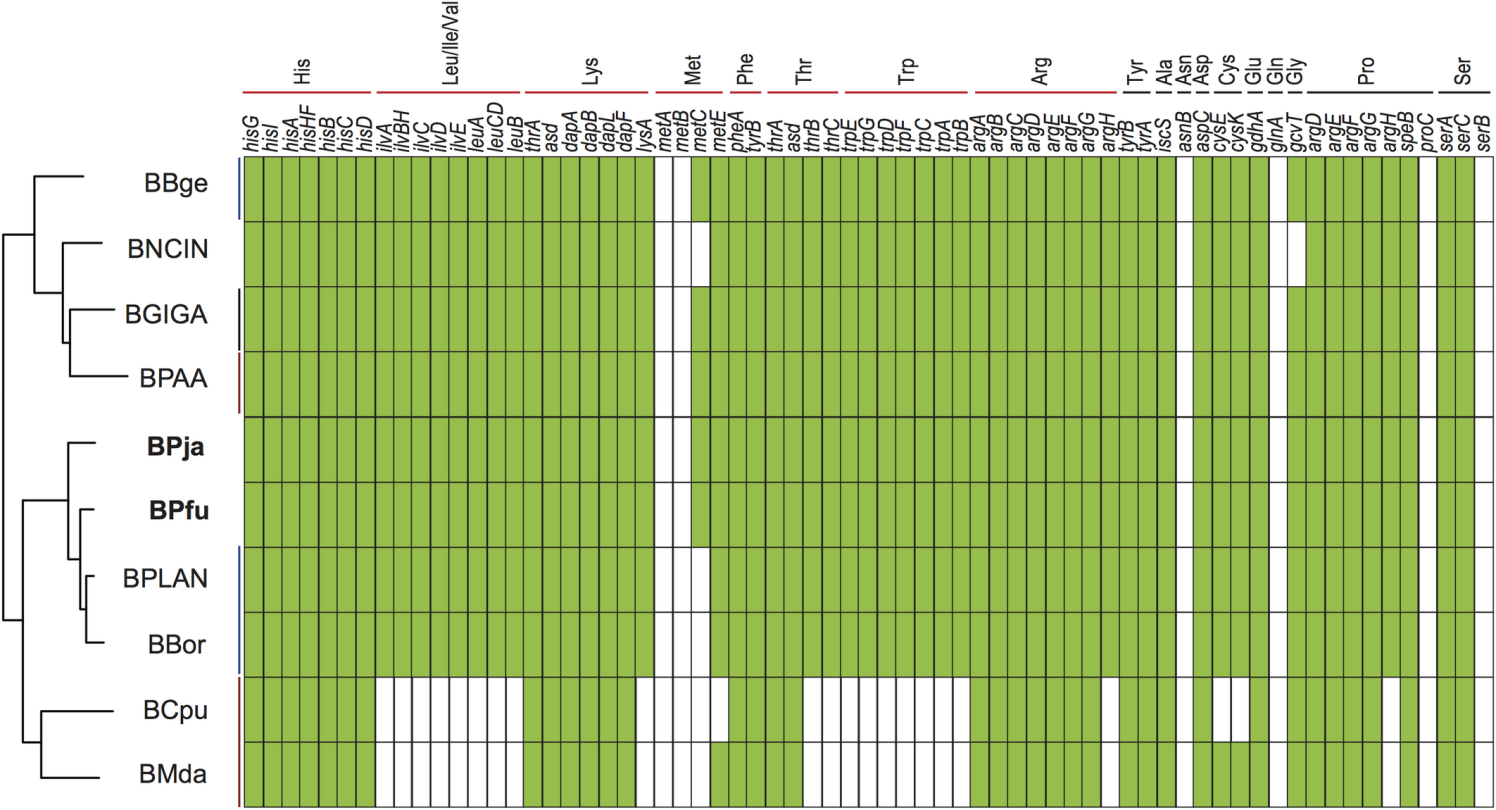
List of genes associated with amino acid synthesis in all sequenced *Blattabacterium* strains. The absent genes are represented by white boxes. Host species abbreviations: BPfu, *Periplaneta fuliginosa*; BPja, *Periplaneta japonica*; BNCIN, *Nauphoeta cinerea*; BGIGA, *Blaberus giganteus*; BBge, *Blattella germanica*; BPLAN, *Periplaneta americana*; BBor, *Blatta orientalis*; BPAA, *Panesthia angustipennis*; BCpu, *Cryptocercus punctulatus*; BMda, *Mastotermes darwiniensis*. Vertical bars indicate omnivorous (blue), wood-feeding (red), and litter-feeding (black) hosts, respectively. EAA are indicated by orange horizontal bars, and non-EAA indicated by black horizontal bars.

Cockroaches are incapable of *de novo* essential amino acid (EAA) synthesis, relying mostly in their diet and in their *Blattabacterium* endosymbionts [2, 8]. The putative metabolic pathway reconstruction from the genome sequence revealed that both BPja and BPfu have the complete biosynthetic pathways for nine EAA (His, Leu, Ile, Val, Lys, Phe, Thr, Trp and Arg) and six non-EAA (Tyr, Ala, Asp, Cys, Glu and Gly) amino acids (Fig 4). The gene set for Asn and Gln biosynthesis is absent in both strains. Whereas only the terminal step enzyme is missing, in case of Pro and Ser biosynthetic pathways. The comparison of amino acid biosynthetic pathways of BPja and BPfu with the other eight-sequenced *Blattabacterium* genomes [8, 10, 12, 14, 16] revealed that none of these strains are able to synthesize all the amino acids. However, the complete biosynthetic pathways of His, Phe, Tyr, Asp, and Glu are present in all *Blattabacterium* strains. All the strains are capable to synthesize all the EAA, except BCpu and BMda that are unable to synthesize Leu, Ile, Val, Thr and Trp.

**Fig 5:**
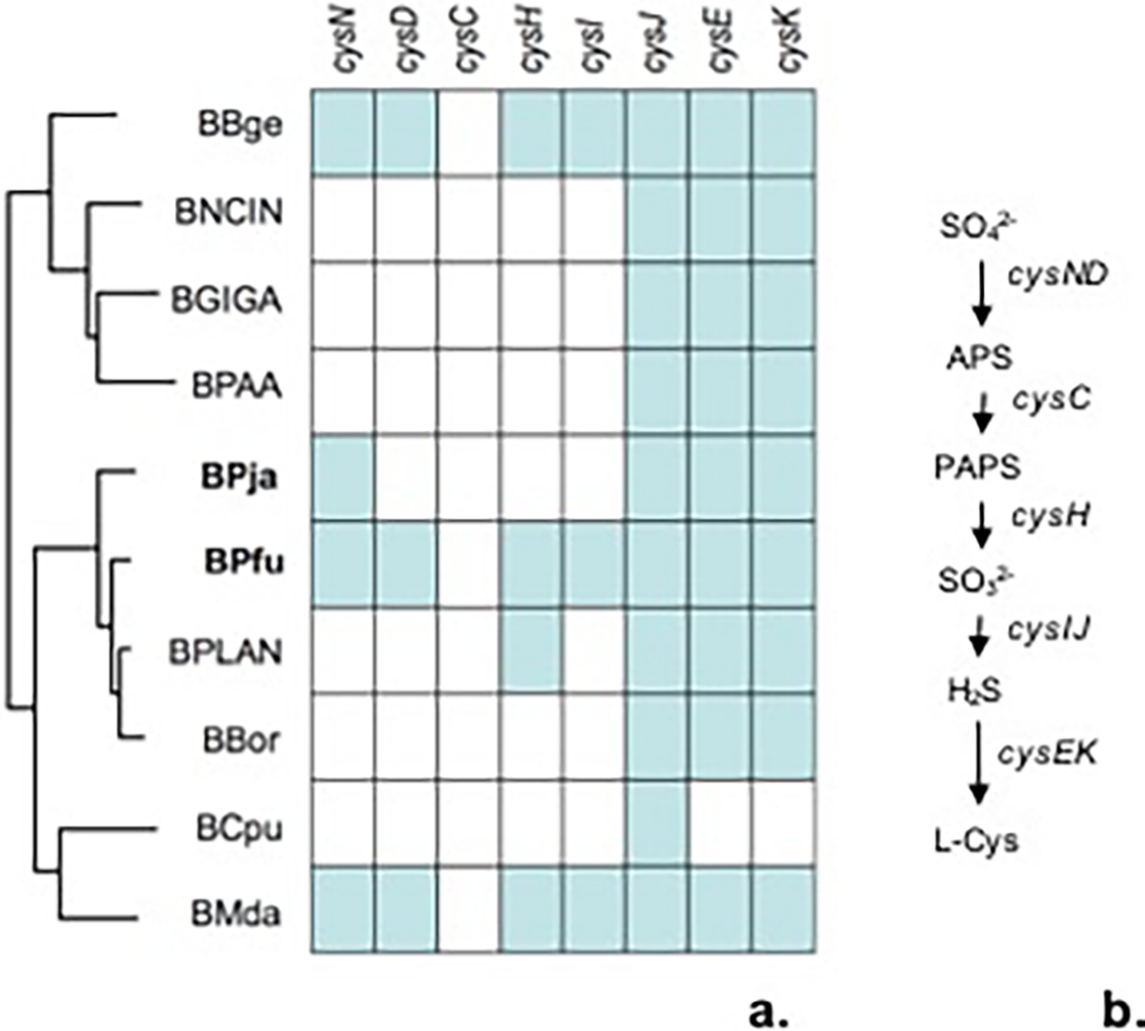
The pathway of sulfate assimilation from different *Blattabacterium* strains. (a). Genes required for sulfur assimilation (b) include *cysN* and *cysD* coding for two subunits of sulfate adenyltransferase; the adenosine 5’-phosphosulfate (APS) reductase *cysH* and the sulfite reductase *cysIJ*. There is a missing step for the conversion of adenosine-5'-phosphosulfate (APS) into 3'-phospho adenosine-5'-phosphosulfate (PAPS). The generated sulfite is reduced to hydrogen sulfide further on assimilated into sulfur-containing amino acids L-cysteine and L-methionine.

Most insect species are unable to use reduce oxidized sulfur compounds and incorporate them into biomolecules, depending thus on their diet or the activity of their endosymbionts [37]. The endosymbiont *Buchnera aphidicola*, the primary endosymbiont of aphids, can assimilate sulfate into sulfur-containing amino acids as Cys or Met [36]. In *Blattabacterium*, only BBge [38, 39] and BMda [15] are able to assimilate sulfate as sulfur donor, possessing a cluster of 7 genes coding for all enzymes involved (*cysNDHIJEK*), in exception of *cysC* (5’-phosphosulfate kinase). Likewise, we found that BPfu also possess all genes for sulfate assimilation like BBge and BMda (Fig 5). In contrast, BPja have lost most of the genes of this pathway (*cysDCHI*).

**Fig 6:**
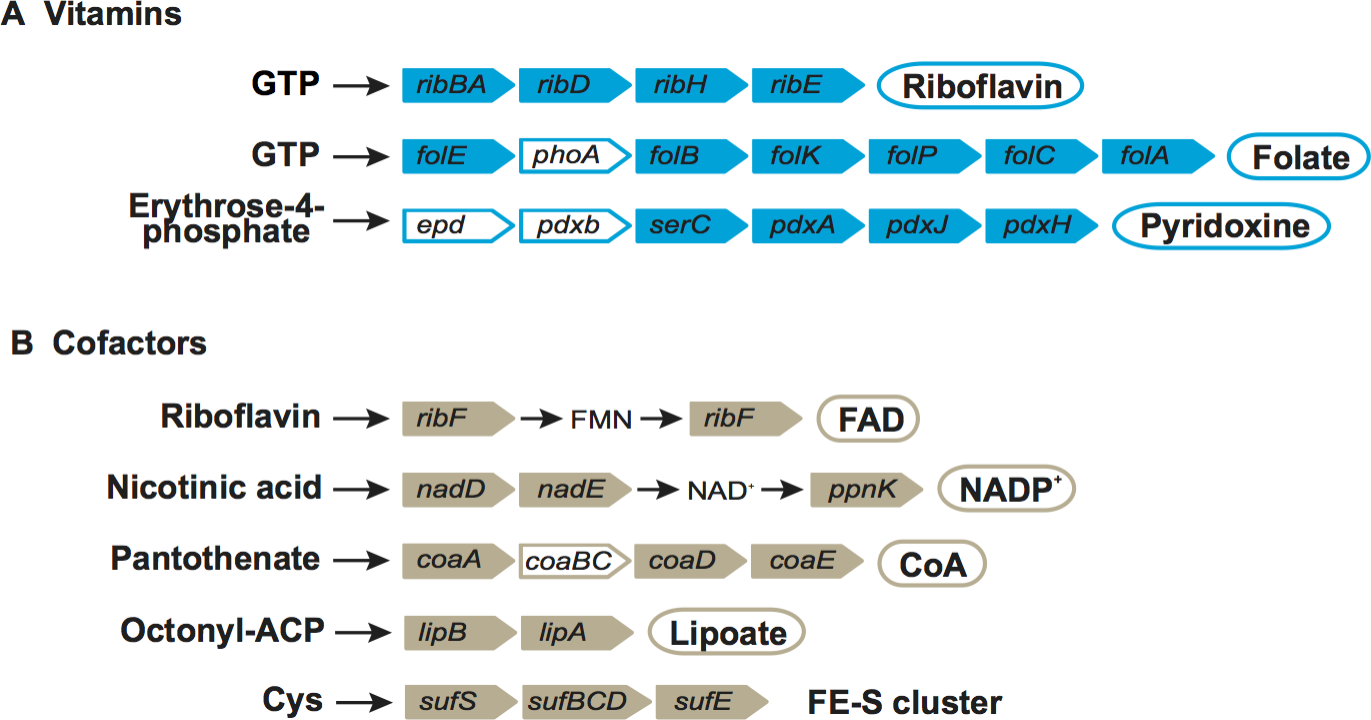
**Reconstruction of pathways for biosynthesis of vitamins (a) and cofactors (b) in *Periplaneta fuliginosa* and *P*. *japonica*.** Gene names are indicated in coloured rectangles. White rectangles indicate missing genes and circles indicate products.

BPja and BPfu are able synthesize some vitamins and cofactors including riboflavin (vitamin B2), pyridoxine (vitamin B6), folate (vitamin B9) and the cofactors FAD, NADP^+^, Coenzyme A, lipoate, FE-S cluster, molybdopterin (Fig 6). Like other endosymbionts, the missing enzymes of vitamin and cofactor biosynthesis might compensate from either the host or from co-occurring endosymbionts [40–41].

## Conclusions

Aside from their ability to live indoors, *P*. *japonica* and *P*. *fuliginosa* are adapted to contrasting environmental conditions being the first native from cooler northern climates and the latter from warmer climates and with low or absent cold-resistance [42–43]. These habitats preferences also impose differences in their diet source, which as in other insect species lead to slight differences in the genome content of their endosymbionts. Although the newly sequenced genomes of BPja and BPful emphasise the remarkable stability of *Blattabacterium* genomes, we were able to detect changes in terms of genome synteny and content. The most surprising result was observed in BPja in the sulfur assimilatory pathway, which is similar to BBge and BMda, endosymbionts of contrasting hosts respectively the cockroach *B*. *germanica* and termite *M*. *darwiniensis*.

## Supporting information

S1 Fig Standard curves for qPCR quantification of *Blattabacterium (ureA)* from *Periplaneta japonica* (a) and *P*. *fuliginosa* (b); and host (*wg*) *P*. *japonica* (c) and *P*. *fuliginosa* (d).
(TIF)

S1 Table Predicted genes in the *Blattabacterium sp*. from *Periplaneta fuliginosa* (BPfu) genome. (XLSX)

S2 Table Predicted genes in the *Blattabacterium sp*. from *Periplaneta japonica* (BPja) genome. (XLSX)

S3 Table Accession number of all genomes used for phylogenomics analysis. (XLSX)

S4 Table Protein-coding genes have been assigned COG numbers for all *Blattabacterium* strains. (XLSX)
