# Supplementary figures and images for: Genome analysis of new Blattabacterium spp., obligatory endosymbionts of Periplaneta fuliginosa and P. japonica
Source: PLoS One. 2018 Jul 10;13(7):e0200512. doi: 10.1371/journal.pone.0200512 (PMC6039017; doi:10.1371/journal.pone.0200512)

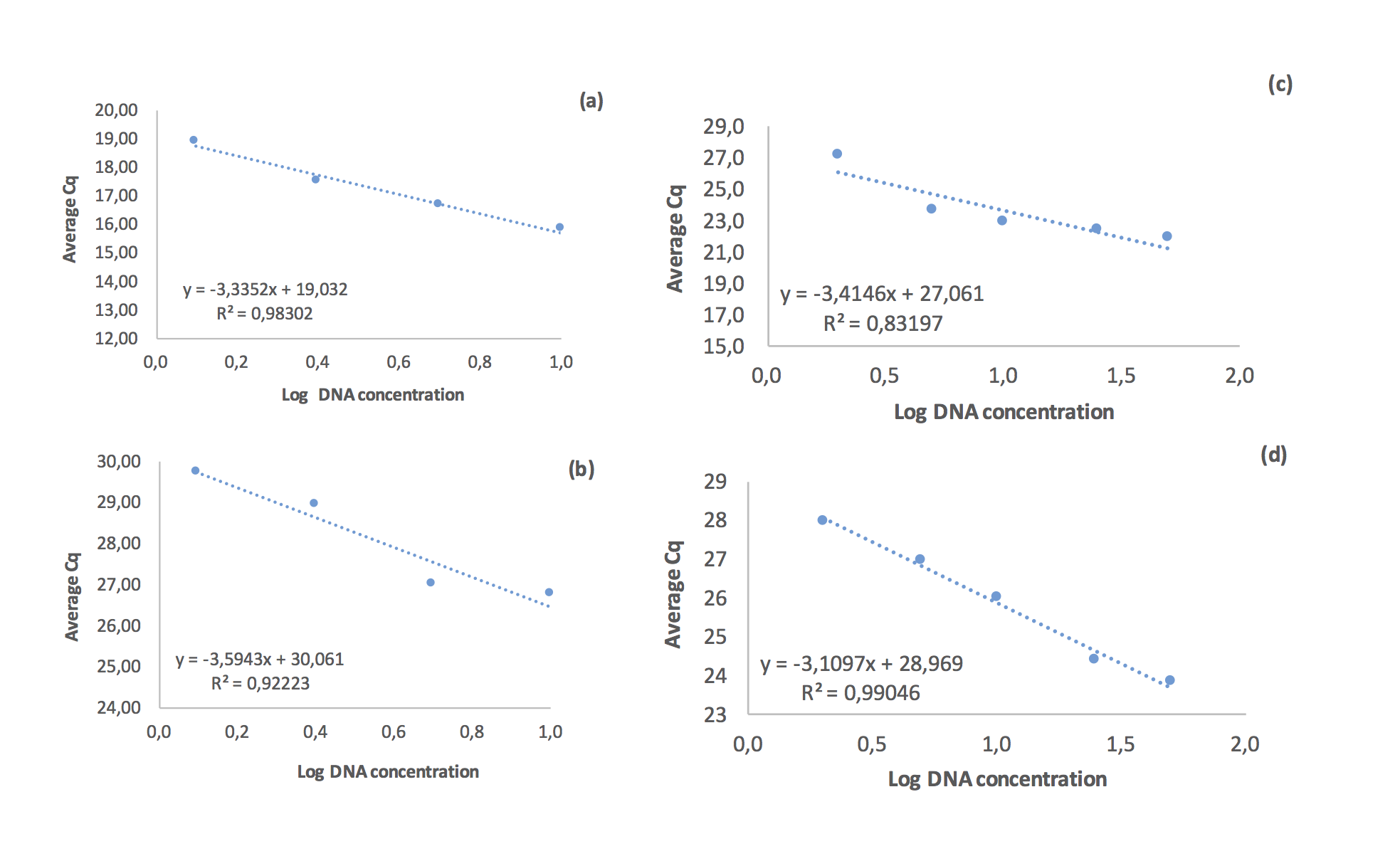

Supplement: S1 Fig — Standard curves for qPCR quantification of Blattabacterium (ureA) from Periplaneta japonica (a) and P. fuliginosa (b); and host (wg) P. japonica (c) and P. fuliginosa (d). (TIF) [file pone.0200512.s001.tif]
